# The Field of Science

**Published:** 1893-10-28

**Authors:** 


### The Field of Science.

It is with a sense of relief that one turns from the statement that no less than 1,790 damaged persons sought relief last year at the Pasteur Institute, to the subsequent sequel which attests that, out of this high figure, five only of the patients succumbed to rabies. And even as regards these small proportion of victims, one must bear in mind that they even might be yet lessened were it not that long delay, owing to distances having to be travelled, is greatly against success in subsequent inoculation. For even the most distant countries produce subjects for anti-rabic vaccination, Algeria especially being mentioned as sending the greatest number of patients for M. Pasteur's treatment.

---

A French contemporary has been discussing in its pages various methods for the disposal of city refuse, a vexed question in large as in small centres. We can be fairly certain, however, that the thrifty mind of the French once given to this subject, it will not be long ere they will evolve some excellent means of converting waste substances into marketable wares. Meanwhile a new experiment in this same direction has been tried in our own country at the Rochdale Sanitary Works, Lancashire. The apparatus here brought into play is worked on the blast system, and converts the combustible matter of the injected refuse into gas, the uncombustible into molten slag. Regarding the latter, it is considered quite possible that this also can be applied to some useful end in the manufacture, for instance, of paving blocks suitable for building purposes.

---

The Indian Commission appointed by the Government for a fuller investigation on leprosy, has now settled the question in the negative as to the hereditary transmission of the disease. This decision, arrived at after a very limited time of research, will find ready combatants in those persons who have given up the best part of their lives to the study of leprosy in its various bearings, and whose experience has not been such as to make them in sympathy with the ultimatum of this august body. The very nature of the complaint is one which demands exclusively a close personal investigation, extending over a number of years, before any opinion on the subject of its heredity can be conclusively formed. It is insufficient to trust too much to the testimony of the afflicted themselves, for to the Hindoo, as to the Eastern mind in general, the very fact that the disease is looked upon as a divine visitation for sin is provocative of a reticence on the part of its victims as to all past family history; therefore it is, as we have said, that a lengthy personal study is manifestly necessary before any less conclusive evidence on the disease can be universally accepted.

The worship of water has been contemporaneous with the existence of man himself, and the varied transitions through which the worship has passed are interesting as illustrative of the march of human intellect, of the gradual evolving of primitive superstition and ignorance into the enlightened days of the nineteenth century, when we find the old idol still bowed down to, but erected on a newer pedestal, and based on a scientific footing. In pre-Christian times, when medical science was in the hands of the priest, suffering humanity turned to those votaries of religion alike for their moral as for their physical cure. The priest who divined the cause of the disease alone could suggest a healing, and the cure was in accord with the "diagnosis," supernatural agencies being prescribed and called into action. In those days our forefathers took their bodily ailments to some brook or well, some water which was peopled by them with mythical deity and presiding genius, whose anger could be assuaged and goodwill bought at the price of a votive offering here presented in the spirit of sacrifice laid down in its twofold order as expiatory and eucharistical. Here it was that the evil spirit, which (according to the priest) molested the suffering body, was extirpated through a tangible vehicle, the unclean mantle was left behind, the body and soul refreshed into a perfect regeneration.

---

To the mind of man in early ages water was surrounded with uncanny influences; its action, they argued, was guided by life and by will, and did not bear in any sense the interpretation of ordinary laws of nature; the worship of the Element was ever a dominant feature in the mind of the Aryan race, an impelling motive based on the firmly rooted belief in its unfailing agency for good. In the fastnesses of the Welsh mountains traditions concerning these sacred spots still hold sway, the aroma of a past romance hanging still about the shrines of a dead and gone worship of past ages. These were early days in the history of curative medicine, the first gropings in the dark after a knowledge which has since assumed less limited environment. The votive offerings of pre-Christian man, the religious ablutions in the days of Moses, are, as has been observed elsewhere, sacred institutions which merely find a secularisation in the hydropathic treatment of to-day. Through the history of mankind it is interesting to trace the uninterrupted sequence regarding the uniformity of veneration in which water has been held, a veneration which has only changed in kind and not in degree. Man's intelligence, as it progressed, replaced the mystic for the divine, the Guardian spirit of the well for the patron saint, until ultimately science assumed supremacy in a domain whereon she now reigns in supreme control.

